# Economic and epidemiological evaluation of text message-based interventions in patients with the Human Immunodeficiency Virus*

**DOI:** 10.1590/1518-8345.3614.3365

**Published:** 2020-09-30

**Authors:** Wendel Mombaque dos Santos, Marcelo Ribeiro Primeira, Larissa Garcia de Paiva, Stela Maris de Mello Padoin

**Affiliations:** 1 Empresa Brasileira de Serviços Hospitalares, Hospital Universitário de Santa Maria, Santa Maria, RS, Brazil.; 2 Universidade Federal de Santa Maria, Departamento de Enfermagem, Santa Maria, RS, Brazil.; 3 Scholarship holder at the Coordenação de Aperfeiçoamento de Pessoal de Nível Superior (CAPES), Brazil.

**Keywords:** HIV, Costs and Cost Analysis, Text Messaging, Controlled Clinical Trial, Cost-Benefit Analysis, Communicable Diseases, HIV, Custos e Análise de Custo, Envio de Mensagens de Texto, Ensaio Clínico Controlado, Análise Custo-Benefício, Doenças Transmissíveis, VIH, Costos y Análisis de Costo, Envío de Mensajes de Texto, Ensayo Clínico Controlado, Análisis Costo-Beneficio, Enfermedades Transmisibles

## Abstract

**Objective:**

to evaluate the cost-effectiveness ratio and the budget impact of sending text messages associated with medical consultations in order to reduce the viral load of patients infected with the Human Immunodeficiency Virus.

**Method:**

a randomized clinical trial, basis for the development of a dynamic cohort model with Markov states in order to compare medical appointments for adults infected with the Human Immunodeficiency Virus *versus* the alternative strategy that associated medical consultations to sending text messages through telephone.

**Results:**

156 adults participated in the study. As for the viral load, it was verified that in the control group there was an increase, in the intervention group A (weekly messages) there was a reduction (p = 0.002) and in group B (biweekly messages) there was no statistically significant difference. Sending text messages would prevent 286,538 new infections by the Human Immunodeficiency Virus and 282 deaths in the 20-year period, compared to the standard treatment. The alternative strategy would result in saving R$ 14 billion in treatment costs.

**Conclusion:**

weekly sending messages in association with the standard treatment can reduce the circulating viral load due to its effect in decreasing new infections, in addition to reducing health costs.

## Abstract (pt)

**Objetivo:**

avaliar a razão custo-efetividade e o impacto orçamentário do envio de mensagens de texto, associadas às consultas médicas, para redução da carga viral de pacientes infectados com o Vírus da Imunodeficiência Humana.

**Método:**

ensaio clínico randomizado, base para o desenvolvimento de um modelo de coorte dinâmica com estados de Markov a fim de comparar consultas médicas para adultos infectados pelo Vírus da Imunodeficiência Humana *versus* a estratégia alternativa que associou consultas médicas, ao envio de mensagens de texto pelo telefone.

**Resultados:**

participaram do estudo 156 adultos. Quanto à carga viral, foi verificado que no grupo controle houve aumento, no grupo intervenção A (mensagens semanais) houve redução (p=0,002) e no grupo B (mensagens quinzenais) não houve diferença estatisticamente significante. O envio de mensagens de texto evitaria 286.538 novas infecções pelo Vírus da Imunodeficiência Humana e 282 mortes no período de 20 anos, ao comparar com o tratamento padrão. A estratégia alternativa resultaria em uma economia de R$ 14 bilhões nos custos de tratamento.

**Conclusão:**

o envio semanal de mensagens em associação ao tratamento padrão pode reduzir a carga viral circulante por seu efeito na diminuição de novas infeções, além da redução de custos em saúde.

## Abstract (es)

**Objetivo:**

evaluar la relación costo-efectividad y el impacto presupuestario del envío de mensajes de texto, asociados con consultas médicas, para reducir la carga viral de pacientes infectados con el Virus de Inmunodeficiencia Humana.

**Método:**

ensayo clínico aleatorizado, que sirve de base para el desarrollo de un modelo de cohorte dinámico con estados de Markov para comparar consultas médicas para adultos infectados con el Virus de Inmunodeficiencia Humana versus la estrategia alternativa que consistió en asociar las consultas médicas al envío de mensajes de texto por teléfono móvil.

**Resultados:**

156 adultos participaron en el estudio. En cuanto a la carga viral, se encontró que en el grupo control hubo aumento, en el grupo de intervención A (mensajes semanales) hubo una reducción (p = 0,002) y en el grupo B (mensajes quincenales) no hubo diferencias estadísticamente significativas. El envío de mensajes de texto podría evitar 286.538 nuevas infecciones por el Virus de Inmunodeficiencia Humana y 282 muertes en el período de 20 años, en comparación con el tratamiento estándar. La estrategia alternativa resultaría en un ahorro de R$ 14 mil millones en costos de tratamiento

**Conclusión:**

el envío semanal de mensajes de forma complementaria al tratamiento estándar puede reducir la carga viral circulante debido a su efecto en la disminución de nuevas infecciones, además de reducir los costos en materia de salud.

## Introduction

Infections caused by the Human Immunodeficiency Virus (HIV) have shown a progressive increase over time since the first identified cases of the disease^(^ 1 ^)^. After the appearance and registration of these cases, dating from the early 1980s, approximately 70 million people contracted the infection and, of these, about 35 million died^(^ 1 ^)^.

Currently, it is estimated that approximately 37 million people live with HIV in the world, characterizing it as one of the main threats to public health, especially in low or middle income countries^(^ 1 ^)^. Despite all the progress made in recent years and the reduction of annual infections by 3% in the period from 2007 to 2017, this infection continues to spread throughout the world, leading to 1.8 million new infections/year and 1 million deaths/year^(^ 1 ^)^.

An example of one of the greatest progresses made in combating this epidemic was the discovery of anti-retroviral treatment (ART) for HIV infection, but its effectiveness and efficiency depend on a series of factors associated with patient compliance. For this reason, the assessment of adherence has been verified by different mechanisms, such as real-time assessment and the verification of the concentration of ART in the hair or blood^(^ 2 ^-^ 4 ^)^. The adoption of measures to assess adherence is essential, as inadequate adherence to ART causes an increase in circulating viral load and, consequently, directly interferes in the control of infection and disease progression^(^ 3 ^-^ 4 ^)^. In addition, there is an impact on the patient’s life expectancy and influence on medical costs related to the progression of the disease, complications, hospitalizations and the treatment of new infections, with adherence being the primary factor for the control of this infection and the early detection of HIV^(^ 3 ^)^.

In Brazil, ART, as we currently know it, was introduced in 1996 with the principles of universal and free access to the health services and medications, in accordance with the Brazilian health system policy^(^ 5 ^-^ 6 ^)^. This policy has achieved good results, mainly in terms of reducing morbidity and mortality, reducing hospitalizations and treatment costs, leading to rates similar to those of developed countries^(^ 5 ^-^ 7 ^)^.

However, this success achieved by Brazilian health policy still faces the poor adherence of patients to ART, which goes beyond free access to treatment, since it depends on the patient’s ability to overcome the barriers that negatively impact their adherence^(^ 5 ^,^ 8 ^-^ 10 ^)^. In this sense, there is a need for investment in actions and strategies that can mitigate the cultural, social, and economic differences of these patients, collaborating in improving the adherence and effectiveness of ART, which must be proposed by health services and supported by public health policies^(^ 1 ^,^ 4 ^,^ 9 ^-^ 11 ^)^.

In recent years, some systematic reviews have shown that interventions based on medical consultations, nursing consultations, telephone calls, text messaging, financial incentives and behavioral therapy have improved adherence to ART, but none has carried out an assessment from an economic and epidemiological perspective, aiming to demonstrate how these interventions interfere in the long term in the incidence of new cases^(^ 3 ^-^ 4 ^,^ 9 ^,^ 12 ^-^ 14 ^)^. In this sense, non-medication and cost-effective interventions, from an economic point of view, such as sending text messages, can help maintain adherence throughout ART without dispensing high costs to the health system and with easy applicability for large populations regardless of their location and facility to the health system^(^ 7 ^,^ 14 ^-^ 15 ^)^. In addition, few studies estimate the impact of these interventions on the population scenario, considering their costs and the possibility of reducing new infections.

Thus, this study aimed to assess the cost-effectiveness ratio and the budget impact of sending text messages associated with medical consultations in order to reduce the viral load of HIV-infected patients. Additionally, the number of new infections prevented and the budget impact were evaluated as a way to propose a cost-effective action to improve adherence and decrease HIV infection rates.

## Method

This is a randomized controlled trial (RCT) that served as a basis for the development of a dynamic cohort model with Markov states to compare the standard treatment (isolated medical visits) for HIV-infected people *versus* the alternative strategy (medical visits and sending text messages). The Markov model is indicated for use in dynamic models of transmission of infectious diseases, since this model is capable of simulating interactions among human beings and how these interactions affect the spread of a disease, HIV in the case of this study, throughout time^(^ 16 ^)^. In addition, this model allows for the inclusion of details relevant to the spread of these diseases, such as different mortality rates, birth rates, and probability of infection according to the severity of the pathology^(^ 16 ^)^.

Text messages are also known for their acronym, SMS (Short Message Service), compatible with practically every mobile phone today. In order to increase the transparency of the economic model of the proposed study, this study was prepared according to the recommendations of the CHEERS Task Force Report checklist^(^ 17 ^)^.

Conducting the RCT was necessary due to the lack of information in the literature regarding the best frequency of sending text messages to reduce viral load in the context of the public health system in Brazil. The prospective and double-blind RCT was performed at the University Hospital of Santa Maria, linked to the Federal University of Santa Maria, from July 2016 to October 2018, in order to assess the impact on the viral load of sending SMS. The referred Hospital assists approximately 1,200 people infected with HIV, of whom 500 are being followed up at the infectious disease outpatient clinic and the others attend the service for laboratory tests and medication withdrawal. This study was approved by the Research Ethics Committee of the Federal University of Santa Maria and published in the Brazilian Registry of Clinical Trials, under the RBR-9nt9hv identifier.

The population was composed of adults infected with HIV and who had been on ART for at least 3 months. The exclusion criteria were the following: presenting some limitation that would make it difficult to understand or express themselves verbally and being in prison, due to the unavailability of access to cell phones.

For the sample calculation, α = 0.05, β = 0.2, q1 = 0.25; q2 = 0.25, q0 = 0.5, P0 = 0.85, and P1 = 0.6 were considered, using the following formula: $A=\mathrm{Za}\sqrt{P\left(1-P\right)\left(1/q1+1/q0\right)}=1.750;\;B=Z\beta \sqrt{P1\left(1-P1\right)\left(1-q1\right)+P0\left(1-P0\right)\left(1/q0\right)}=0.722;\;C=\left(P1-P0\right)2=0.063;\;\mathrm{Total}\;\mathrm{Pop}.=N=\left(A+B+C\right)2/C=147.$. When the formula was applied, a minimum total population of 147 participants was obtained. The selection of participants was carried out in a randomized way by simple draw.

The interventions were carried out according to the following groups:

- Control group – They received reminders of consultations with an infectious disease physician containing date and time and monthly check for receipt of messages.
- Intervention group A – They received reminders of consultations with an infectious disease physician containing date and time, monthly check for receipt of messages, and a weekly social support text message.
- Intervention group B – They received reminders of consultations with an infectious disease physician with date and time, monthly check for messages, and a biweekly social support text message.

The monthly check for the receipt of text messages consisted of sending a message every 30 days, in which the patient should answer this message with a requested number and letter. This confirmation aims to ensure that the study participants were still receiving and reading the messages that were sent to them.

There was no group with daily and monthly messages, based on studies that showed that these sending frequencies are less effective than the weekly or biweekly ones, and the text messages used in the intervention groups are based on the conceptual framework of social support^(^ 12 ^,^ 18 ^-^ 19 ^)^. The messages sent did not inform the diagnosis of HIV infection, respecting the patients’ privacy. The messages were sent in the following order based on social support domains and, at the end of the last message (number 6), it returned to the initial message (number 1)^(^ 19 ^)^:

1. Keep strong. We at the Santa Maria University Hospital care and take care of you.
2. Everyone feels sad at times. Remember that you can talk about depression at your next appointment.
3. Smile, breathe, and go ahead.
4. Invest in your health. Remember to eat healthy foods and practice physical activities.
5. Be active in your health. Keep your appointments scheduled.
6. Have you taken your medication? They help, even if you think they are not working.

Data collection was carried out by applying a questionnaire to characterize the population of adults infected with HIV, which occurred at 2 different moments: Moment 1 – Questionnaires for characterization of the population and viral load (month 0); Moment 2 – Viral load (month 6).

Data analysis was performed by means of a descriptive analysis of the variables and multiple linear regression in order to assess the hazard ratio for the reduction of viral load values, considering viral suppression (viral load below 50 copies/ml).

The dynamic cohort model was based on a hypothetical cohort of 210,659,013 individuals in Brazil in 2019, of which 827,000 were infected with HIV, being studied for a period of 20 years. The analysis was performed in the TreeAge Pro 2019r software (TreeAge Software Inc., Williamstown, MA).

The model was based on HIV infection prevalence rates in Brazil considering the year 2017^(^ 1 ^,^ 20 ^)^. The 20-year period was chosen to demonstrate the HIV infection chain, in which it is important to keep infected people on viral suppression to avoid the risk of transmitting the virus. In this scenario, possible outbreaks of infection were not considered.

**Figure 1:**
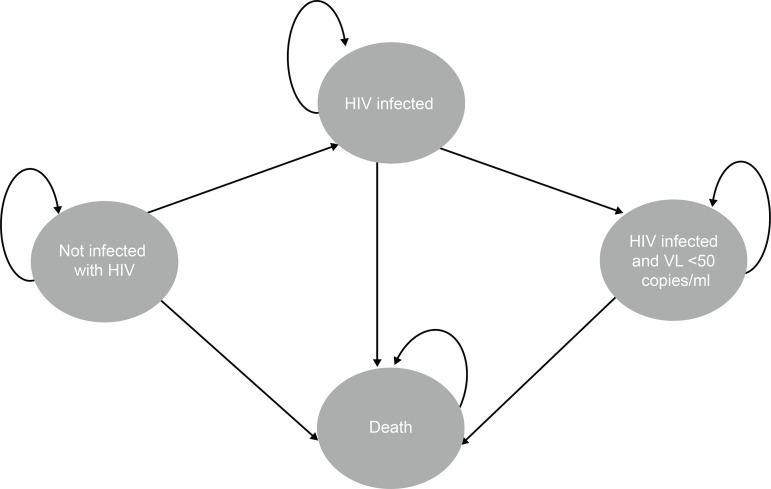
Markov Model

The analysis compared two strategies: the standard treatment provided by the Unified Health System (*Sistema* Único *de Saúde*, SUS) and an alternative strategy with the inclusion of sending text messages to the standard treatment (the most effective sending frequency). According to the model diagram (Figure 1), the treatment strategy considers four transition states, which are changed in annual cycles:

- People not infected with HIV, that is, are susceptible to infection. At each cycle they may remain uninfected, acquire HIV infection or evolve to death.
- People infected with HIV and with a viral load above 50 copies/ml, that is, are the people who have the possibility of transmitting the virus. At each cycle they can remain infected, evolve to undetectable viral load or evolve to death.
- People undergoing treatment and with an undetectable viral load (viral load below 50 copies/ml), that is, they have HIV, but do not have the possibility of transmitting the virus. At each cycle they can have detectable viral load or evolve to death.
- People who die, which may or may not be related to the infection situation. People who are in this cycle do not have the possibility to change to another state of health.

The economic analysis was carried out from the perspective of the SUS. The perspective of the public health system included direct medical costs (medical consultation, medication, laboratory tests, and hospitalizations due to complications). The costs were estimated in reais, with reference to the year 2018 (US$ = 1.00 - R$ 3.89). Future costs and effectiveness were discounted at 5% *per* year.

The effectiveness data were based on data from the RCT (reduction of viral load) and on data from the literature (general infection rate)^(^ 21 ^-^ 24 ^)^. In the model, an estimate was made that, in 2018, approximately 90% of the HIV-infected patients were aware of their serological condition^(^ 5 ^)^.

The cost data were estimated on the cost of sending a weekly text message for a period of 1 year, the costs of treating people with an undetectable viral load, and the cost of treating people with a detectable viral load. The cost of the treatment considered the possibility of hospitalizations, as well as the greater or lesser need for laboratory tests.

The multivariate analysis verified the impact of the set of variables on the model, which was performed by Monte Carlo simulation (100,000 interactions), which randomly chooses values from the parameter distributions to jointly estimate the costs and effects of each strategy.

The variables and values used to estimate the parameters for the distributions were described in Table 1.

**Table 1:** Model parameters. Santa Maria, RS, Brazil, 2018

Parameter	Value
Initial population - Not infected with HIV	210,659,013
Initial population - HIV infected	285,150
Initial population - Infected with HIV and viral load below 50 copies/ml	541,850
Initial population - Dead	0
Time horizon*	20
Cycles per year	1
Annual mortality rate - Not infected with HIV	0.00647
Annual mortality rate - HIV infected	0.0066284
Annual mortality rate - Infected with HIV and viral load below 50 copies/ml	0.006518
Natality	0.01461
Discount rate	0.05
Utility - Not infected with HIV	1.00
Utility - HIV infected	0.50
Utility - Infected with HIV and viral load below 50 copies/ml	0.70
Infection rate^†^	0.15
Annual cost^‡^ - HIV infected	7,430.56
Annual cost^‡^ - Infected with HIV and viral load below 50 copies/ml	3,867.75
Annual cost^‡^ - Weekly SMS	12.00
Effectiveness - SMS^§^	1.2683
Effectiveness - Standard treatment	0.101030524

* In years;

† Beta distribution;

‡ Value expressed in reais;

§ Hazard ratio

## Results

Initially, 168 individuals participated in the study; however, only 156 completed the RCT. Among the individuals who did not complete the research, 1 withdrew, 1 died, and 10 did not show up for final data collection, resulting in 51 participants in the control group, 53 in intervention group A, and 52 in intervention group B. They were predominantly female (57.1%; n = 89), lived with a spouse or a partner (51.3%; n = 80), and HIV infection occurred through sexual transmission (69.9%; n = 109).

**Figure 2:**
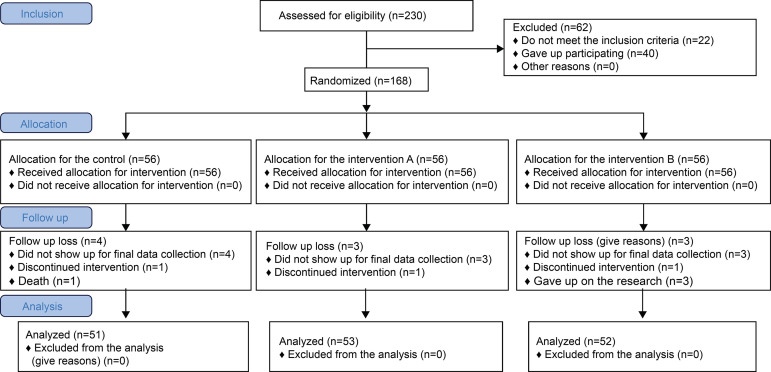
Flowchart of the randomized clinical trial

Regarding the circulating viral load values according to the groups, it was verified that, in the control group, there was an increase in the studied period (pre viral load = 2,638.85; post viral load = 11,890.31; p = 0.001), in intervention group A (weekly SMS sending) there was a reduction in the studied period (pre viral load = 4,598.92; post viral load = 5.68; p = 0.002) and, in group B (biweekly SMS sending) there was no statistically significant difference in the studied period (pre viral load = 854.11, post viral load = 3.367,83; p = 0.649). Thus, sending weekly SMSs was 1.26 times more likely to have a viral load below 50 copies/ml when compared to the group not submitted to intervention.

The dynamic cohort model estimated that, with the standard treatment in the 2019 cohort, 29,386,767 people would die and 2,011,964 would be infected with HIV in the 20-year period. These cases would result in R$ 141 billion in total treatment costs. The alternative strategy (SMS) would avoid 286,538 new HIV infections and 282 deaths in the 20-year period when compared to the standard treatment. The alternative strategy would result in saving R$ 14 billion in treatment costs (Table 2).

**Table 2:** Comparison between standard and SMS treatment over 20 years. Santa Maria, RS, Brazil, 2018

Year	Population without HIV infection		Population with HIV infection		Population with HIV infection and undetectable viral load		Deaths total population		Cumulative cost	
	Current Scenario	SMS Scenario	Current Scenario	SMS Scenario	Current Scenario	SMS Scenario	Current Scenario	SMS Scenario	Current Scenario	SMS Scenario
2019	209,832,013	209,832,013	285,150	285,150	541,850	541,850	-	-	4,214,564,522	4,224,488,522
2020	211,513,910	211,513,910	298,465	291,572	565,731	572,624	1,358,635	1,358,635	8,616,136,229	8,611,886,617
2021	213,208,094	213,209,124	312,395	298,133	590,736	603,970	2,728,360	2,728,359	13,213,248,604	13,165,116,514
2022	214,914,599	214,917,752	326,968	304,834	616,918	635,902	4,109,265	4,109,262	18,014,815,697	17,887,162,131
2023	216,633,457	216,639,894	342,214	311,680	644,330	668,432	5,501,442	5,501,437	23,030,148,567	22,781,070,170
2024	218,364,698	218,375,650	358,161	318,673	673,029	701,574	6,904,983	6,904,974	28,268,972,369	27,849,951,221
2025	220,108,348	220,125,121	374,843	325,815	703,074	735,342	8,319,980	8,319,967	33,741,444,138	33,096,980,889
2026	221,864,430	221,888,408	392,292	333,110	734,526	769,750	9,746,528	9,746,509	39,458,171,271	38,525,400,929
2027	223,632,965	223,665,613	410,541	340,561	767,451	804,811	11,184,721	11,184,694	45,430,230,742	44,138,520,407
2028	225,413,968	225,456,838	429,629	348,171	801,916	840,541	12,634,654	12,634,618	51,669,189,075	49,939,716,870
2029	227,207,453	227,262,187	449,590	355,943	837,991	876,953	14,096,423	14,096,376	58,187,123,086	55,932,437,535
2030	229,013,430	229,081,763	470,465	363,880	875,750	914,064	15,570,124	15,570,065	64,996,641,437	62,120,200,499
2031	230,831,904	230,915,671	492,295	371,986	915,270	951,888	17,055,856	17,055,782	72,110,907,005	68,506,595,958
2032	232,662,876	232,764,016	515,121	380,264	956,630	990,442	18,553,716	18,553,625	79,543,660,111	75,095,287,449
2033	234,506,344	234,626,904	538,987	388,717	999,915	1,029,740	20,063,804	20,063,694	87,309,242,618	81,890,013,107
2034	236,362,299	236,504,441	563,940	397,349	1,045,213	1,069,800	21,586,219	21,586,088	95,422,622,933	88,894,586,939
2035	238,230,731	238,396,734	590,026	406,164	1,092,614	1,110,638	23,121,063	23,120,908	103,899,000,000	96,112,900,115
2036	240,111,621	240,303,891	617,297	415,164	1,142,214	1,152,271	24,668,436	24,668,254	112,756,000,000	103,549,000,000
2037	242,004,947	242,226,021	645,804	424,354	1,194,112	1,194,715	26,228,443	26,228,230	122,009,000,000	111,207,000,000
2038	243,910,683	244,163,232	675,599	433,736	1,248,412	1,237,989	27,801,185	27,800,939	131,677,000,000	119,090,000,000
2039	245,828,793	246,115,635	706,741	443,316	1,305,223	1,282,110	29,386,767	29,386,484	141,778,000,000	127,204,000,000

From the SUS point of view, the alternative strategy (SMS) is dominant (lower cost and greater effectiveness) compared to the standard treatment. SMS showed a cost-effectiveness ratio of R$28.24 and the standard treatment R$ 31.48, at the population level.

**Figure 3:**
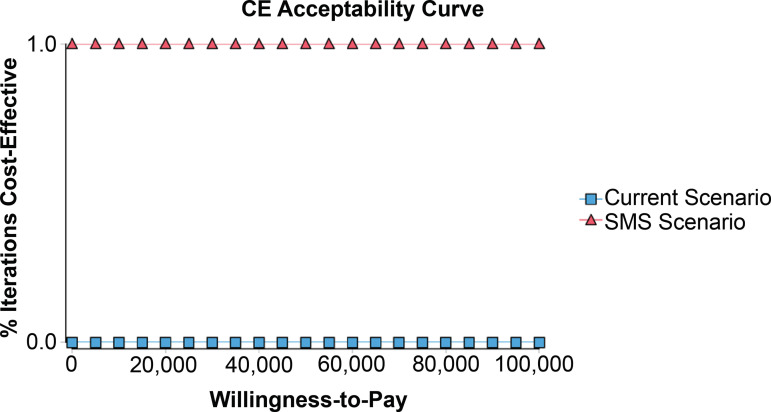
Probabilistic sensitivity analysis

The probabilistic sensitivity analysis indicated that, regardless of the willingness to pay, the alternative strategy (SMS) is the most economical intervention when compared to the standard treatment (Figure 3).

## Discussion

This study verified that, compared to the usual clinical practice, the addition of weekly SMS sending was economically more effective in reducing the circulating viral load of patients infected with HIV and on ART. Within the period studied, the weekly SMS intervention increased by 26% the chance of the patient having an undetectable viral load. Despite the additional cost of the SMS intervention for each research subject, a long-term saving was verified, as shown in the economic impact study, due to the reduction in the incidence of HIV infections.

The SMS intervention generates savings of nearly R$ 14 billion in terms of treatment costs for the SUS in Brazil and helps in the reduction of 263,424 new HIV infections, considering the 20-year period. When projecting the effects of the discrepancy at the beginning of treatment between the two groups, for the same period of life, we verified that the SMS-based intervention accumulated more years of life gain (incremental effectiveness) and lower cost (incremental cost) than the control group. Maintaining the standard treatment would result in an additional cost of R$ 50,862.36 per infection when compared to the SMS intervention. The robustness of the lifetime model was verified by the sensitivity analysis, with variations of the transition probabilities and costs. The SMS intervention was the dominant treatment (lowest cost and highest effectiveness) compared to the current treatment (highest cost and lowest effectiveness), which leads to a negative Incremental Cost-effectiveness Ratio (ICER).

The results found in this study are similar to those of other analyses regarding savings in interventions to improve adherence to treatment, which consider the feasibility of screening in the population, nursing interventions, and connectivity through an application to clarify and control patients with HIV^(^ 25 ^-^ 28 ^)^. Our conclusions are consistent with the arguments that even interventions with modest effects can be more cost-effective compared to only the usual care and treatments^(^ 25 ^-^ 28 ^)^. These indicators demonstrate that the adoption of additional health care strategies may enhance the maintenance of adherence in medium to long term.

Approaches based on sending SMS, as shown in this study, may improve adherence to ART; however, there is no consensus in the literature regarding the content of SMS, particularly with regard to the open discussion of the exposure of patients under treatment. Thus, this study used indirect messages or reminders, used as a means for initiating contact or for reminding the participants about the treatment, similarly to research carried out in developing countries. The adoption of these measures contributes to the patient’s perception of the support offered by the health team, corroborating the maintenance of adherence^(^ 8 ^,^ 14 ^,^ 18 ^-^ 19 ^)^.

Sending SMS has been significantly used in the health area, mainly to improve quality of life and attendance in primary care, and to reduce non-adherence and improve health results at low cost, as well as the possibility of wide dissemination of information in real time for the entire target population of the intervention^(^ 8 ^,^ 13 ^-^ 14 ^,^ 18 ^-^ 19 ^,^ 29 ^)^. Additionally, the use of SMS reminders is an accessible, adequate, and more cost-effective tool compared to those already spent on medication^(^ 26 ^,^ 30 ^-^ 31 ^)^. In this way, there are basically four types of benefits of interventions performed by SMS: (1) improved efficiency in the provision of health care; (2) improvement in treatment adherence; (3) public health benefits; (4) low cost and good accessibility^(^ 19 ^,^ 26 ^,^ 28 ^,^ 30 ^-^ 31 ^)^.

The main strength of this dynamic cohort analysis was that the sources of the intervention parameter (sending SMS, and control group) were based on real-life data, from an RCT. In addition, the model estimated the evolution of the intervention over 20 years, demonstrating the linear increase in cost over this period, and the sensitivity analysis demonstrated the robustness of the predicted model. It is worth highlighting that this is the first study carried out in the context of the SUS that considers an intervention based on SMS at the population level.

This study had some limitations. First, the effectiveness of the SMS intervention was based on the RCT data, but some clinical and epidemiological data were obtained from the Ministry of Health data source; however, the values of these parameters were applied to both groups (SMS *versus* control), thus not affecting the relative effectiveness of the model. Second, the parameters of this study were derived from a 6-month intervention, which may have diluted the effect of the SMS intervention in the population context over time, just as with each year of planned intervention there is an increase in data uncertainty. However, these limitations do not preclude the results presented since, in the first year after the intervention, positive results are already obtained.

The results presented in this study demonstrate an advance in knowledge through the use of an RCT associated with a dynamic cohort model with Markov states, of the long-term evolution of interventions in patients with HIV, demonstrating its impact on transmissibility, mortality, and costs. Therefore, this study may subsidize complementary health actions through other technologies of communication with the patient which contribute to the increase of adherence in patients on ART and to a consequent reduction of transmissibility, as well as allowing for its replication in different contexts.

## Conclusion

The analysis of this dynamic cohort reflects that, when implemented at the population level, weekly sending SMS to people infected with HIV and on ART can reduce the circulating viral load and lead to a consequent reduction of new infections, in addition to the reduction of direct costs related to treatment in health.
